# A Rare Case of Neonatal-Onset Diarrhea Due to Congenital Tufting Enteropathy: Clinical, Histopathological, and Genetic Insights

**DOI:** 10.3390/reports9030310

**Published:** 2026-09-12

**Authors:** Maria Rogalidou, Konstantina Dimakou, Kalliopi Stefanaki, Amalia Patereli, Alexandra Papadopoulou

**Affiliations:** 1 Division of Gastroenterology, Hepatology & Nutrition, First Department of Pediatrics, Medical School, National & Kapodistrian University of Athens, “Agia Sofia” Children’s Hospital, 11527 Athens, Greece; 2 Department of Gastroenterology, “Agia Sofia” Children’s Hospital, 11527 Athens, Greece; 3 Pathology Department, “Agia Sofia” Children’s Hospital, 11527 Athens, Greece

**Keywords:** congenital tufting enteropathy, EPCAM, congenital diarrhea, intestinal failure, infant, failure to thrive, total parenteral nutrition

## Abstract

**Background and Clinical Significance:** Congenital tufting enteropathy (CTE), or autosomal recessive congenital diarrhea type 5 (DIAR5), is a rare intestinal epithelial disorder characterized by severe neonatal-onset watery diarrhea, failure to thrive, and intestinal failure. Most cases are caused by biallelic EPCAM variants. **Case Presentation:** A 53-day-old female infant presented with severe watery diarrhea and failure to thrive from the first days of life. She was born at term with a birth weight of 3.4 kg and had 10–20 watery stools daily. At admission, she was dehydrated, dystrophic, and below the third weight percentile. Diarrhea persisted despite an amino acid-based formula and cessation of enteral feeding. Duodenal biopsy showed disturbed villous–crypt architecture, variable lamina propria inflammation, absence of intraepithelial lymphocytosis, epithelial tufts, and regenerative epithelial changes, supporting CTE. Whole-exome sequencing identified compound heterozygous EPCAM variants: a maternally inherited likely pathogenic frameshift variant, c.687_688del, p.(Lys230Asnfs*23), and a paternally inherited missense variant, c.757G>A, p.(Asp253Asn), classified as a variant of uncertain significance. TPN was initiated for intestinal failure. Follow-up was complicated by catheter-related sepsis, cholelithiasis, and recurrent pancreatitis requiring laparoscopic cholecystectomy. She subsequently tolerated limited enteral feeding but did not achieve enteral autonomy and remained dependent on TPN, while maintaining normal growth with nutritional support. **Conclusions:** CTE should be considered in infants with severe neonatal-onset diarrhea. Histopathology combined with genetic testing is essential for diagnosis. Long-term TPN and multidisciplinary management are required.

## 1. Introduction and Clinical Significance

Congenital diarrheal disorders (CDDs) are a heterogeneous group of rare intestinal diseases characterized by persistent diarrhea beginning during the neonatal period or early infancy. They result from defects in intestinal digestion, absorption, epithelial integrity, immune regulation, or transport mechanisms. Diagnosis can be challenging because manifestations overlap with common causes of infantile diarrhea (Table 1), including infectious enteritis, cow’s milk protein allergy, and metabolic disorders. Delayed diagnosis may result in dehydration, malnutrition, growth failure, and intestinal failure requiring nutritional support [1,2,3,4].

Congenital tufting enteropathy (CTE; OMIM #613217), also known as autosomal recessive congenital diarrhea type 5 (DIAR5), is a rare epithelial enteropathy characterized by severe watery diarrhea, failure to thrive, and frequent dependence on long-term total parenteral nutrition (TPN). It is mainly caused by biallelic pathogenic variants in the EPCAM gene, which encodes epithelial cell adhesion molecule (EpCAM), a protein involved in epithelial integrity and intestinal barrier function [7]. Histologically, CTE is characterized by epithelial tufts and variable villous abnormalities. Genetic testing is increasingly important for diagnosis, particularly in atypical cases [3,5,8].

Although approximately 150 patients with CTE had been reported by 2020, and subsequent studies have further expanded the molecular characterization of EPCAM-associated disease [9], CTE remains an important diagnostic consideration in infants presenting with severe neonatal-onset diarrhea. We report a female infant with severe neonatal-onset diarrhea, characteristic histological findings, and compound heterozygous EPCAM variants, including a likely pathogenic frameshift variant and a missense variant of uncertain significance (VUS).

## 2. Case Presentation

A 53-day-old female infant was referred for evaluation of persistent diarrhea and failure to thrive. Profuse watery diarrhea without blood or mucus had been present since the first days of life, with 10–20 watery stools daily.

She was the first child of healthy, non-consanguineous parents and was born at term after an uncomplicated pregnancy and delivery, with a birth weight of 3.4 kg. At admission, she was dehydrated and had a dystrophic appearance. Her weight remained unchanged from birth and was below the third percentile, whereas length and head circumference were at the 50th percentile. Physical examination revealed no dysmorphic features or abnormalities from other systems.

Initial investigations, including complete blood count, serum biochemistry, immunological studies, virological testing, and peripheral blood immunophenotyping, were unremarkable. Blood and stool cultures were negative for bacterial pathogens, and stool testing for adenovirus, rotavirus, and parasites was negative.

**Figure 1:**
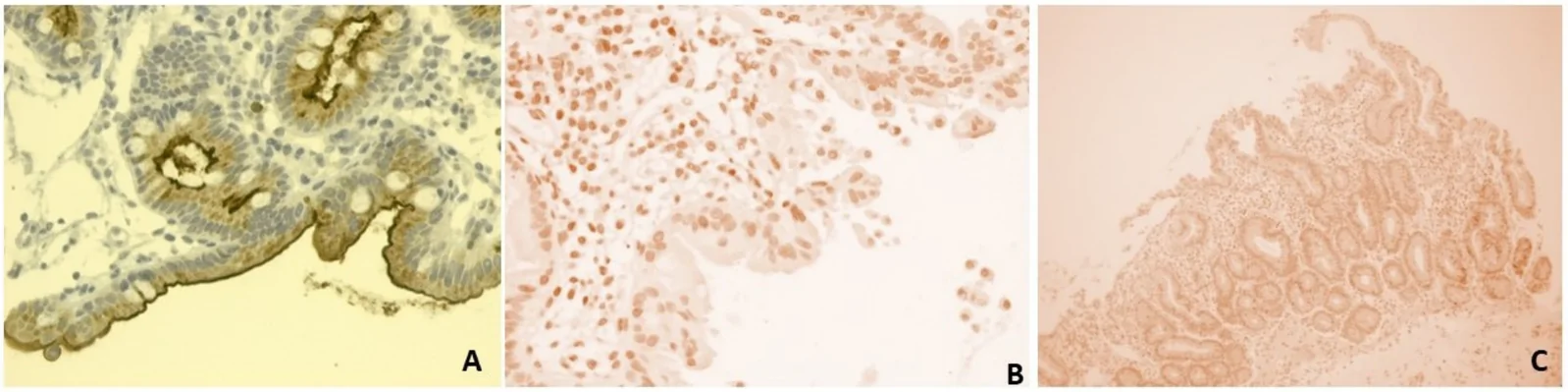
Histological findings of congenital tufting enteropathy (CTE). (**A**): Histological examination of the duodenal mucosa demonstrated disturbed villous–crypt architecture, variable inflammation of the lamina propria, and absence of significant intraepithelial lymphocytosis. (**B**): Characteristic focal epithelial tufts were observed, accompanied by regenerative epithelial changes. (**C**): Immunohistochemical staining showed an abnormal/absent epithelial membrane antigen (Ep-CAM/MOC-31) pattern, supporting the diagnosis of congenital tufting enteropathy. These findings, in conjunction with the clinical presentation and exclusion of other causes of persistent diarrhea, support CTE as the underlying intestinal disorder in the present case.

Diarrhea persisted despite discontinuation of enteral feeding, and previous treatment with an amino acid-based formula produced no improvement. Upper gastrointestinal endoscopy showed no macroscopic abnormalities. Duodenal biopsy specimens demonstrated normal maltase and isomaltase activity. Rectosigmoidoscopy with no relevant findings and cytomegalovirus PCR was negative. Histopathological examination of the duodenal mucosa demonstrated disturbed villous–crypt architecture, variable lamina propria inflammation, absence of intraepithelial lymphocytosis, and characteristic epithelial tufts with regenerative epithelial changes, suggesting CTE (Figure 1).

Whole-exome sequencing identified compound heterozygous EPCAM variants: a maternally inherited frameshift variant, c.687_688del, p.(Lys230Asnfs*23), classified as likely pathogenic, and a paternally inherited missense variant, c.757G>A, p.(Asp253Asn), classified as a VUS. The clinical presentation, histopathological findings, and molecular results supported the diagnosis of EPCAM-related congenital tufting enteropathy (DIAR5).

Because of severe intestinal failure, a central venous catheter was inserted and TPN was initiated. Continuous nasogastric feeding was introduced simultaneously to provide enteral stimulation. Initially, the patient was completely dependent on parenteral nutrition.

During the subsequent clinical course, the patient developed several complications during prolonged parenteral nutrition and central venous access. At 14 months of age, she experienced an episode of catheter-related sepsis, which was treated with appropriate antibiotic therapy, with subsequent clinical resolution. At 19 months of age, she developed a central venous catheter-associated Staphylococcus aureus bacteremia, for which the Hickman catheter was removed and subsequently replaced. The bacteremia resolved following appropriate antimicrobial treatment. She subsequently developed cholelithiasis. Despite treatment with ursodeoxycholic acid, she developed an episode of pancreatitis followed by three recurrent episodes. Given the recurrent pancreatitis in the setting of cholelithiasis, laparoscopic cholecystectomy was performed at 5 years of age. Following cholecystectomy, no further episodes of pancreatitis occurred.

Over time, she became able to receive a limited amount of enteral feeding primarily for oral stimulation and social interaction. However, this limited enteral intake did not result in a meaningful reduction in her parenteral nutrition requirements. At the most recent follow-up, she maintained normal growth parameters but remained predominantly dependent on TPN, with only limited enteral intake.

At the most recent follow-up, one month prior to manuscript submission, at 7 years and 4 months of age, the patient remained dependent on TPN but maintained normal growth parameters. Her weight was 27 kg (82nd percentile; Z-score +0.92), height was 128 cm (83rd percentile; Z-score +0.95), and BMI was 16.48 kg/m^2^ (71st percentile; Z-score +0.55). Her neurodevelopmental assessment was appropriate for age. She continued to receive a limited amount of enteral nutrition, primarily for oral stimulation and social interaction, without a meaningful reduction in her parenteral nutrition requirements.

## 3. Discussion

CTE is a rare but well-documented cause of severe, persistent neonatal-onset diarrhea. Approximately 150 patients with CTE had been reported in the literature by 2020 [8]. Subsequent case reports and genetic studies have further expanded the clinical and molecular characterization of this disorder [10]. More recently, a systematic review of EPCAM-associated CTE identified 150 molecularly confirmed cases [11]. Our case adds to the existing literature by describing a female infant with severe neonatal-onset diarrhea, characteristic histopathological findings, and compound heterozygous EPCAM variants, including a likely pathogenic frameshift variant and a missense variant of uncertain significance (VUS).

CTE is a rare autosomal recessive congenital enteropathy characterized by severe, persistent, neonatal-onset watery diarrhea, intestinal failure, and variable degrees of growth impairment. Most cases are associated with biallelic pathogenic variants in EPCAM [3,5,8]. The diagnosis can be challenging because the clinical presentation overlaps with other congenital diarrheal disorders, including microvillus inclusion disease, congenital chloride diarrhea, glucose–galactose malabsorption, and immune-mediated enteropathies [2,3,12]. Early recognition is important because prolonged diarrhea and intestinal failure may result in severe nutritional deficiencies and prolonged dependence on parenteral nutrition.

In our patient, diarrhea began shortly after birth and persisted despite discontinuation of enteral feeding and dietary interventions. The severe intestinal failure, failure to thrive, and persistent diarrhea were suggestive of an underlying congenital enteropathy. Duodenal biopsy demonstrated disturbed villous–crypt architecture, variable lamina propria inflammation, absence of significant intraepithelial lymphocytosis, and characteristic epithelial tufts with regenerative epithelial changes. These histopathological findings, together with the clinical presentation and exclusion of other major causes of persistent neonatal diarrhea, strongly supported the diagnosis of CTE. Although characteristic epithelial tufting remains an important diagnostic feature, histopathological findings may overlap with other enteropathies and should therefore be interpreted in conjunction with clinical and molecular findings [3,5,8]. Genetic testing is increasingly important for molecular confirmation and may also help distinguish CTE from phenotypically similar congenital diarrheal disorders.

EpCAM is a transmembrane epithelial cell adhesion molecule that plays an important role in epithelial adhesion, cell–cell interactions, intestinal barrier integrity, and epithelial organization. Impaired EpCAM function can disrupt epithelial architecture and barrier function, resulting in intestinal dysfunction and the characteristic phenotype of CTE [6,7]. Biallelic EPCAM variants are associated with the typical form of CTE, which generally manifests predominantly as intestinal disease. In contrast, biallelic SPINT2 variants have been associated with a syndromic form of congenital tufting enteropathy accompanied by extraintestinal abnormalities, particularly choanal or anal atresia and ophthalmologic manifestations [9].

Whole-exome sequencing in our patient identified compound heterozygous EPCAM variants: a maternally inherited truncating variant, c.687_688del, p.(Lys230Asnfs*23), classified as likely pathogenic, and a paternally inherited missense variant, c.757G>A, p.(Asp253Asn), classified as a variant of uncertain significance (VUS). The clinical significance of p.(Asp253Asn) therefore requires careful interpretation. Importantly, this variant has previously been reported in association with congenital tufting enteropathy. In a previous study [10], a patient homozygous for the p.(Asp253Asn) variant was reported, with characteristic epithelial tufting demonstrated on histopathological re-evaluation. The same variant has also been reported in association with absent intestinal EpCAM expression, suggesting a potential functional effect. The variant is potentially relevant because it is present in trans with a likely pathogenic EPCAM variant in a patient with a highly characteristic CTE phenotype, including neonatal-onset persistent diarrhea, severe intestinal failure, and characteristic epithelial tufting on duodenal biopsy. The concordance between the clinical and histopathological phenotype and the identification of biallelic EPCAM variants raises the possibility that p.(Asp253Asn) contributes to the disease phenotype.

Nevertheless, the classification of p.(Asp253Asn) as a VUS indicates that the available evidence is currently insufficient to establish its pathogenicity. The presence of the variant in a patient with CTE, even in trans with a likely pathogenic allele, cannot by itself demonstrate that the missense change is deleterious. Accordingly, the molecular findings should not be interpreted as definitive evidence that p.(Asp253Asn) is pathogenic. Rather, the diagnosis in the present case is supported by the characteristic clinical presentation and histopathological findings together with the identification of biallelic EPCAM variants, one likely pathogenic and the other of uncertain significance. Functional studies evaluating the effect of p.(Asp253Asn) on EpCAM expression, cellular localization, protein function, or epithelial integrity would be particularly valuable. Additional segregation studies and identification of unrelated individuals with the same variant and a compatible phenotype could also provide evidence for future variant reclassification.

The clinical course of our patient further illustrates the severity and chronic nature of CTE. Because of severe intestinal failure, a central venous catheter was inserted and TPN was initiated. Continuous nasogastric feeding was introduced simultaneously to provide enteral stimulation. Initially, the patient was completely dependent on parenteral nutrition. Over time, she became able to tolerate a limited amount of enteral feeding, primarily for oral stimulation and social interaction. However, this limited enteral intake did not result in a meaningful reduction in her parenteral nutrition requirements. She currently maintains normal growth parameters and tolerates some enteral feeding but remains dependent on TPN. This clinical course is consistent with the severe intestinal phenotype reported in patients with CTE and highlights the importance of individualized nutritional management and continued monitoring of intestinal adaptation.

Treatment of CTE remains largely supportive and focuses on nutritional rehabilitation, management of intestinal failure, prevention of complications, and preservation of enteral feeding whenever tolerated [1,5,9]. Long-term TPN may be necessary to maintain adequate growth and development in patients with severe intestinal failure but is associated with substantial morbidity, including catheter-related bloodstream infections, thrombosis, and intestinal failure-associated liver disease. Whenever feasible, even limited enteral nutrition may be beneficial for maintaining oral and gastrointestinal stimulation, although the extent to which enteral feeding can reduce parenteral nutrition requirements varies considerably among patients.

This case highlights the diagnostic value of integrating clinical, histopathological, and molecular findings in patients with severe neonatal-onset persistent diarrhea. The characteristic epithelial tufting and clinical phenotype strongly support CTE, while the identification of compound heterozygous EPCAM variants provides important molecular context. However, because one allele, p.(Asp253Asn), remains classified as a VUS, its individual contribution to the phenotype cannot currently be established. Further functional and genetic evidence will be necessary to clarify the pathogenicity of this variant. This distinction is important for accurate interpretation of the molecular findings and emphasizes the need for cautious genotype–phenotype correlation in rare congenital enteropathies.

## 4. Conclusions

This case expands the clinical and molecular spectrum of EPCAM-related congenital tufting enteropathy by describing compound heterozygous variants consisting of a likely pathogenic frameshift variant and a missense VUS. It emphasizes the diagnostic value of characteristic epithelial tufts combined with molecular testing in infants with severe neonatal-onset diarrhea. Early diagnosis is essential for nutritional management, prevention of complications, genetic counseling, and long-term multidisciplinary care.

## Figures and Tables

**Table 1:** Major causes of diarrhea in neonates and infants [5,6]. Differential diagnosis of persistent diarrhea in the present case. This table summarizes the principal infectious, feeding-related, allergic, congenital, epithelial, pancreatic, immune-mediated, anatomical, metabolic, secretory, and medication-related causes considered during the diagnostic evaluation. The clinical and diagnostic characteristics listed for each category highlight features that may help distinguish these conditions from the phenotype observed in the present case. The persistent nature and clinical characteristics of the patient’s diarrhea, together with the exclusion of common acquired causes, prompted evaluation for an underlying congenital or genetic disorder.

Category	Examples	Typical Clinical/Diagnostic Characteristics
**Infectious**	Viral, bacterial, and parasitic gastroenteritis	Acute diarrhea, fever, vomiting, positive stool microbiology
**Feeding-related**	Overfeeding, inappropriate formula preparation, feeding intolerance	Relationship with feeding; improvement after adjustment of feeding regimen
**Cow’s milk protein allergy**	Non-IgE-mediated cow’s milk protein allergy, food-protein-induced enteropathy	Diarrhea, vomiting, blood/mucus in stool, feeding intolerance
**Congenital diarrheal disorders**	Congenital tufting enteropathy, microvillus inclusion disease, congenital chloride diarrhea, glucose–galactose malabsorption	Neonatal-onset persistent diarrhea, dehydration, failure to thrive, intestinal failure
**Carbohydrate malabsorption**	Glucose–galactose malabsorption, congenital sucrase–isomaltase deficiency	Watery acidic stools, abdominal distension, symptoms related to carbohydrate intake
**Intestinal epithelial disorders**	Congenital tufting enteropathy, microvillus inclusion disease	Severe persistent diarrhea, epithelial/villous abnormalities, TPN dependence
**Pancreatic insufficiency**	Cystic fibrosis, congenital pancreatic insufficiency	Steatorrhea, poor growth, increased fecal fat, low fecal elastase
**Immune-mediated enteropathy**	Autoimmune enteropathy, immunodeficiency-associated enteropathy, very-early-onset IBD	Chronic diarrhea, infections, autoimmune manifestations, abnormal immune findings
**Anatomical/intestinal failure**	Short bowel syndrome, intestinal atresia, extensive intestinal resection	Malabsorption following congenital or acquired intestinal loss; prolonged nutritional support
**Metabolic disorders**	Galactosemia and other inherited metabolic disorders	Diarrhea with vomiting, poor feeding, lethargy, hypoglycemia, liver or systemic abnormalities
**Other secretory disorders**	Congenital enteric endocrine/secretory disorders	Persistent high-volume diarrhea with electrolyte disturbances
**Medication-related**	Antibiotic-associated diarrhea and osmotic agents	Temporal association with medication exposure

Bold for emphasis in different domains.

## Data Availability

The original contributions presented in this study are included in the article. Further inquiries can be directed to the corresponding author.

## Abbreviations

The following abbreviations are used in this manuscript:

CDDs	Congenital diarrheal disorders
CTE	Congenital tufting enteropathy
DIAR5	Autosomal recessive congenital diarrhea type 5
TPN	Total parenteral nutrition
VUS	Variant of uncertain significance
PCR	Polymerase chain reaction.
